# Meetings of Societies

**Published:** 1926

**Authors:** 


### Meetings of Societies.

### Bristol Medico-Chirurgical Society.

*February 10th*, 1926.

Mr. T. Carwardine, President, in the Chair.

Cases and specimens were shown by members of the Society.

Mr. A. Rendle Short and Dr. A. D. Fraser read a paper on "**Unexpected Deaths in the Post-Operative Period.**"

---

*March 10th*, 1926.

Mr. T. Carwardine, President, in the Chair.

Mr. H. D. Gillies read a paper on "**The Functional and Æsthetic Results of Plastic Surgery.**"

Starting with the deformities resulting from gunshot wounds of the face, he stated the broad principles underlying their repair :—

1. Conserve every scrap of undamaged tissue ; with this end in view, close the wound as soon as possible, sewing skin to skin or mucous membrane.

2. Replace all normal tissue in its proper place.

3. Bring fresh healthy tissue to close gaps, both in skeleton and soft parts.

He then spoke of the evolution of the tube graft, and its application in the repair of shell wounds, giving numerous illustrations from photographs of the various stages and the results. The method of measuring and fitting grafts by the help of plaster casts and stencils was described. The use of Thiersch and Wolff grafts was shown, and the occasional cosmetic disappointments with the latter mentioned. When repairing the face, and especially the nose, the lecturer emphasised the necessity of providing a mucous membrane, or at least skin, to replace the destroyed mucous lining of cavities. In this connection he described the " stent " inlay graft, and illustrated its application in the nose, in the mouth, and for ectropion. Loss of part of the lower jaw was repaired by means of bone grafts, and dental prostheses ; cartilage grafts were used to repair nose and forehead, and dental prostheses for loss of the upper jaw. The results of treatment of scarring after severe burns were illustrated.

Mr. Gillies then spoke of the application of these principles to the deformities of civil life resulting from burns, industrial or motor accidents, and extensive operations. The method of treating facial nævi and hairy moles was shown, and the important social and psychic bearing of these deformities mentioned. The general principles of plastic surgery evolved for the treatment of the face was not confined to this region. Destruction of tissue in the limbs might be amenable to treatment along these lines when other methods offered little prospect of success. Means for improving the functional results after cleft palate operations were shown, and the repair of damage to the pudenda in both male and female described. Finally, purely cosmetic manœuvres, such as the rectification of a "Semitic" nose, were indicated. The lecturer concluded by saying that the secret of success was a correct diagnosis; one must ascertain exactly what was missing and had to be restored. Care on the part of the surgeon and patience both from him and from his patient were the other requisites. A very complete series of photographs illustrated each of the various processes, and demonstrated the truly remarkable results that the lecturer has been able to achieve.

---

*April 14th*, 1926.

Mr. T. Carwardine, President, in the Chair.

Dr. Nixon showed a series of cases illustrating his paper on "**Rigid Rest in the Treatment of Rheumatic Heart Disease,**" reported on page 96 of this issue.

Mr. E. Watson-Williams showed cases operated on for the relief of suppurative meningitis, described in the course of his paper on page 91.

Dr. Parker read notes on a "**Case of Uveo-Parotitic Polyneuritis,**" with commentary on reported cases, reported on page 73. The discussions are reported with the papers to which they relate.

The May meeting of the Society was cancelled on account of the General Strike.

